# Compression loading-induced stress responses in intervertebral disc cells encapsulated in 3D collagen constructs

**DOI:** 10.1038/srep26449

**Published:** 2016-05-20

**Authors:** Wai Hon Chooi, Barbara Pui Chan

**Affiliations:** 1 Tissue Engineering Laboratory, Department of Mechanical Engineering, The University of Hong Kong, Pokfulam Road, Hong Kong Special Administrative Region, China

## Abstract

Cells protect themselves from stresses through a cellular stress response. In the interverebral disc, such response was also demonstrated to be induced by various environmental stresses. However, whether compression loading will cause cellular stress response in the nucleus pulposus cells (NPCs) is not well studied. By using an *in vitro* collagen microencapsulation model, we investigated the effect of compression loading on the stress response of NPCs. Cell viability tests, and gene and protein expression experiments were conducted, with primers for the heat shock response (HSR: HSP70, HSF1, HSP27 and HSP90), and unfolded protein response (UPR: GRP78, GRP94, ATF4 and CHOP) genes and an antibody to HSP72. Different gene expression patterns occurred due to loading type throughout experiments. Increasing the loading strain for a short duration did not increase the stress response genes significantly, but over longer durations, HSP70 and HSP27 were upregulated. Longer loading durations also resulted in a continuous upregulation of HSR genes and downregulation of UPR genes, even after load removal. The rate of apoptosis did not increase significantly after loading, suggesting that stress response genes might play a role in cell survival following mechanical stress. These results demonstrate how mechanical stress might induce and control the expression of HSR and UPR genes in NPCs.

Cells protect themselves from stresses in the environment such as heat, ultraviolet light, toxins, pH and oxidative stress through a cellular stress response^1^. When cells encounter a stress stimulus lower than the critical threshold, then they respond to the stress by triggering the appropriate protective responses. These mainly involve a heat shock response (HSR) and unfolded protein response (UPR), which usually take place in the cytoplasm and endoplasmic reticulum (ER), respectively. Mechanical loading may be another environmental stressor to cells especially for the cells that are found in the load bearing tissues. Hence, musculoskeletal cells such as chondrocytes, tendon fibroblasts and pre-osteoblasts, were shown to respond to mechanical stress by upregulating the expression of heat shock proteins (HSPs)^2^,^3^,^4^,^5^,^6^,^7^,^8^. Similarly, in cartilage tissues and osteocytes, ER stress markers were upregulated in response to mechanical stress^9^,^10^,^11^.

In the intervertebral disc (IVD), nucleus pulposus cells (NPCs) are exposed to hostile environments with stressors such as low pH, low nutrients, hypoxia, osmotic pressure and mechanical load. Several studies have demonstrated cellular stress response due to these environmental stressors. For example, HSP27 and HSP72 have been reported to be found in the pathological discs^12^,^13^. Furthermore, rat NPCs upregulated HSP70 in response to hypoxia and increasing osmotic pressure in a dose-dependent manner^14^,^15^. In addition to that, stretching was also demonstrated to induce ER stress in rat annulus fibrosus cells, with enhanced expression of ER stress markers such as glucose regulated protein-78 (GRP78) and C/EBP homologous protein (CHOP)^16^,^17^. However, whether mechanical loading in particular compression will cause cellular stress response including HSR and UPR in the NPCs has not been investigated.

We hypothesize that compression loading stimulates stress responses in NPCs. Specifically, we used a 3D collagen culture model to investigate the effect of different strains, durations and types of compression loading on the cellular stress response, particularly of the HSR and UPR. The 3D collagen culture system was previously shown to be able to preserve the morphology and phenotype of rabbit NPCs^18^.

## Results

### Cell viability

**Figure 1:**
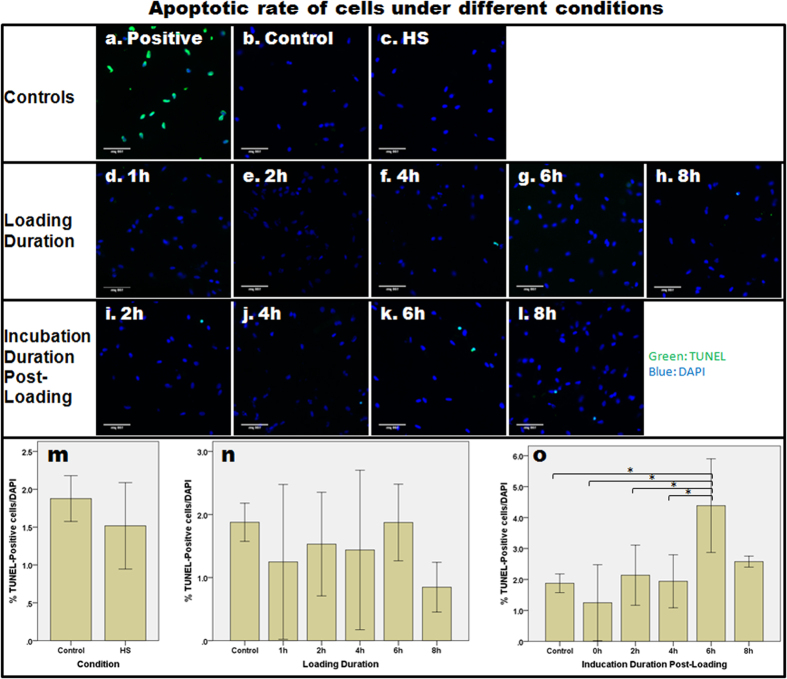
The relative rate of apoptosis in NPCs subjected to different loading durations and incubation durations post-loading was determined using the TUNEL assay. (**a**) In the positive control, apoptotic cells were stained with fluorescein and therefore appeared green in colour. (**b–i**) The rate of apoptosis after static loading with 70% strain with different loading and incubation durations was assessed. (**m–o**) Bar charts to show the mean ± 2SE level of apoptosis (indicated by % TUNEL-positive cells/DAPI-positive cells). Asterisks indicate statistically significant results (p < 0.05).

The live/dead cell viability assay showed that cell viability was maintained under different conditions of loading and after loading (Supplementary Fig. S1). Moreover, in order to study whether loading induced NPCs to become apoptotic, the TUNEL assay was conducted on samples under a high strain of static loading (70% strain) with different loading and incubation durations. Figure 1 shows the percentage of TUNEL-positive cells under the various different conditions. In general, a very low number of TUNEL-positive cells (1–5%) was observed under the different conditions. Immediately after loading, there was no increase in the percentage of apoptotic cells, when compared with the control (one-way ANOVA, p = 0.450). However, the percentage of apoptotic cells was found to be significantly different at the various post-loading incubation times (one-way ANOVA, p = 0.003). Bonferroni’s *post hoc* test showed that the percentage of TUNEL-positive cells at 6-h incubation post-loading was significantly higher than in the control group (p = 0.021), as well as immediately (p = 0.002), 2 h (p = 0.037) and 4 h (p = 0.043) after loading. Nevertheless, the percentage difference among all groups (of <5%) was practically insignificant.

### Gene expression

#### Effect of encapsulation in the collagen construct

**Figure 2:**
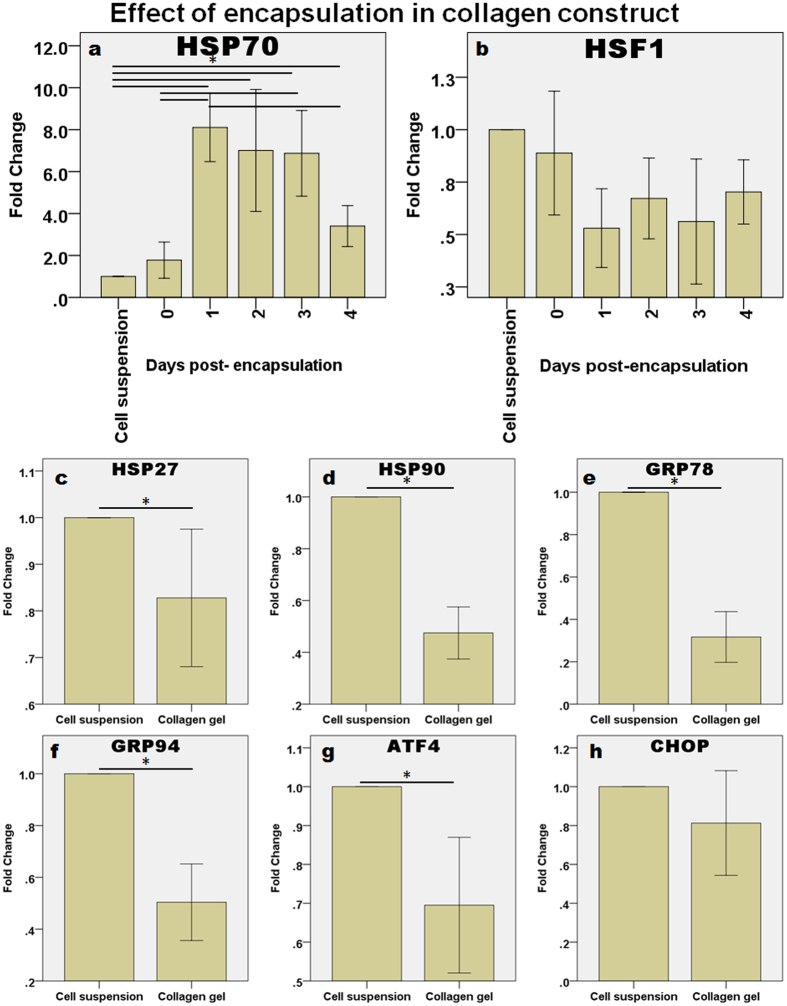
Effect of encapsulation in a collagen construct on the expression of cellular stress response genes. (**a,b**) The expression levels (in fold change) of HSP70 and HSF1 were measured from 0 to 4 days post-encapsulation (n = 6 for each). (**c–h**) The other stress response genes: (**c**) HSP27, (**d**) HSP90, (**e**) GRP78, (**f**) GRP94, (**g**) ATF4, and (**h**) CHOP were downregulated after 4 days of encapsulation in collagen gel. In each case, the mean ± 2SE fold change of gene expression was normalized to 18S rRNA and plotted relative to cell suspension. Asterisks indicate statistically significant differences (p < 0.05). n = 10 for cell suspension and n = 30 for the collagen constructs.

The NPCs might have encountered environmental stress when they were changed from being in monolayer culture to being encapsulated in a collagen construct. Figure 2 shows that the expression of HSP70 was upregulated significantly when the cells were encapsulated (Fig. 2a). Upregulation in HSP70 was the highest one day after encapsulation and then it started to decrease from Day 2 onwards. One-way ANOVA showed that the duration of encapsulation was a significant factor affecting the expression of HSP70 (p < 0.001) but not HSF1 (p = 0.068). Dunnett’s T3 *post hoc* test revealed that HSP70 expression was significantly different from the control at day 1 (p = 0.003), day 2 (p = 0.046), day 3 (p = 0.020) and day 4 (p < 0.001) after encapsulation. The expression of HSP70 was also significantly increased at days 1 and 3, when compared with day 0 (p = 0.002 and 0.028, respectively). Finally, the expression of HSP70 on day 4 dropped significantly when compared with that of day 1 (p = 0.010), suggesting that the encapsulation-induced stress might be transient. The expression of other common stress response genes was also analyzed in samples at day 4. In contrast to HSP70, which, as mentioned, was upregulated when compared with the control on day 4, HSP27 (Fig. 2c), HSP90 (Fig. 2d), GRP78 (Fig. 2e), GRP94 (Fig. 2f), ATF4 (Fig. 2g) and CHOP (Fig. 2h) were all, in general, downregulated. An independent samples *t*-test showed that the downregulation was statistically significant for HSP27 (p = 0.027), HSP90 (p < 0.001), GRP78 (p < 0.001), GRP94 (p < 0.001) and ATF4 (p = 0.002).

#### Effect of heat shock (HS)

A positive control was used to confirm the ability of bovine NPCs to upregulate both heat shock response (HSR) genes (Supplementary Fig. S2a–d) and unfolded protein response (UPR) genes (Supplementary Fig. S2e–h) following heat shock (HS) at 42 °C for one hour. After HS, the expression of all the genes was upregulated except HSF1 (Supplementary Fig. S2b) and GRP78 (Supplementary Fig. S2e), which did not change.

#### Effect of loading strain and frequency

**Figure 3:**
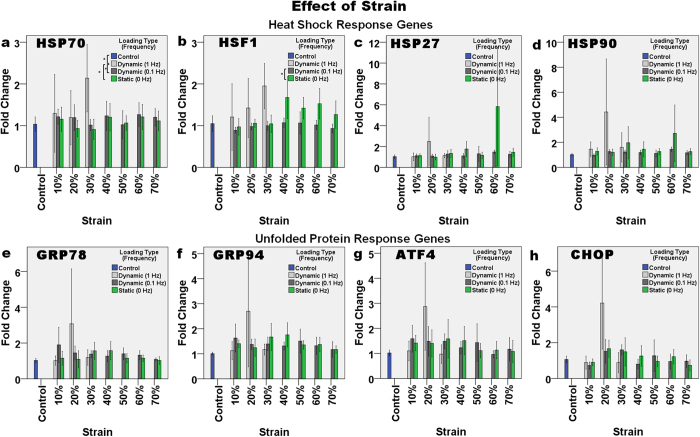
The effect of strain (10–70%) and frequency of compressive loading (1 Hz, 0.1 Hz and 0 Hz) on the relative level of gene expression. The HSR genes: (**a**) HSP70, (**b**) HSF1, (**c**) HSP27, and (**d**) HSP90, and UPR genes: (**e**) GRP78, (**f**) GRP94, (**g**) ATF4, and (**h**) CHOP were only upregulated slightly, after compressive loading for one hour. Asterisks indicate statistically significant differences (p < 0.05). In each case, the mean ± 2SE fold change of gene expression was normalized to the control without loading. n = 10 (control); n = 4 (1 Hz); n = 6 (0.1 Hz) and n = 8 (0 Hz).

The expression of HSR and UPR genes was affected by both the loading strain and loading type, but the effect was mild. Increasing the loading strain in the short term did not seem to increase the stress response. The expression of HSP70 (Fig. 3a) was significantly different among groups with different loading types (p = 0.002). High-frequency (1 Hz) dynamic loading significantly affected HSP70 expression, when compared with the control (p = 0.026), static loading (p = 0.006) and low frequency loading (p = 0.036). When comparing the expression of HSF1 (Fig. 3b), in the control and static loading groups, the latter appeared to show a slightly increased level of HSF1 expression at loading strains >30% but these increases were not significant (p > 0.05). However, two-way ANOVA showed that there was a significant difference in HSF1 expression due to the different loading strains (p = 0.011) and frequencies (p < 0.001). Dunnett’s T3 *post hoc* test after one-way ANOVA showed that only the expression of HSF1 at 0.1 Hz dynamic loading was significantly different from that at static loading (p = 0.001).

#### Effect of loading duration (different loading types)

**Figure 4:**
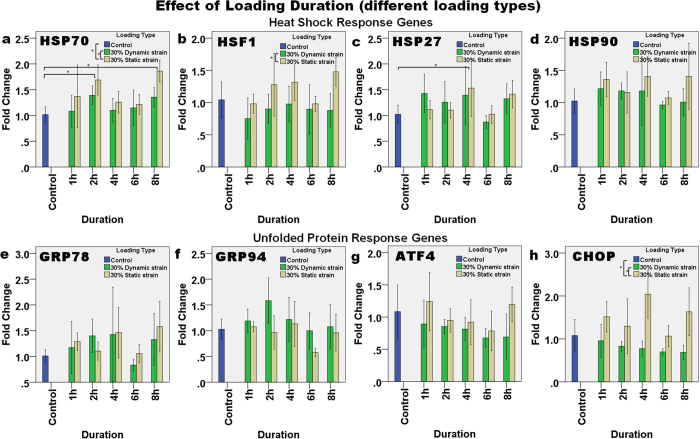
The effect of loading duration and type of compressive loading (dynamic and static) on the relative level of gene expression. The HSR genes: (**a**) HSP70, (**b**) HSF1, (**c**) HSP27, and (**d**) HSP90, and UPR genes: (**e**) GRP78, (**f**) GRP94, (**g**) ATF4, and (**h**) CHOP demonstrated different expression patterns for the different loading type. Asterisks indicate statistically significant differences (p < 0.05). In each case, the mean ± 2SE fold change of gene expression was normalized to the control without loading. n = 3 for each sample.

At 30% strain, the effect of dynamic and static loading at different loading durations was compared (Fig. 4). Only the expression of HSP70 and HSP27 significantly changed with loading duration. For HSP70 (Fig. 4a), a significant difference was found in the expression due to the loading duration (two-way ANOVA, p = 0.009) with an almost two-fold upregulation after 2 h and 8 h of loading (Bonferroni’s *post hoc* test, p = 0.013 and p = 0.004, respectively). On the other hand, for HSP27 expression (Fig. 4c), a loading duration of 4 h had a significant effect on expression (p = 0.041), and Bonferroni’s *post hoc* test showed that this upregulation was significantly different from the control (p = 0.040). The type of loading also significantly affected the expression of HSP70 (two-way ANOVA, p = 0.008), as upregulation due to static loading was significantly higher than that as a result of dynamic loading (p = 0.023) and the control (p = 0.001). Similarly, for expression of HSF1 (Fig. 4b), two-way ANOVA showed that loading type caused significant differences (p = 0.004) and Bonferroni’s *post hoc* test showed significant differences between dynamic and static loading (p = 0.012). The expression of CHOP was also significantly different after static loading than it was after dynamic loading (p < 0.001) and the control (p = 0.054).

#### Effect of loading durations (different strains)

**Figure 5:**
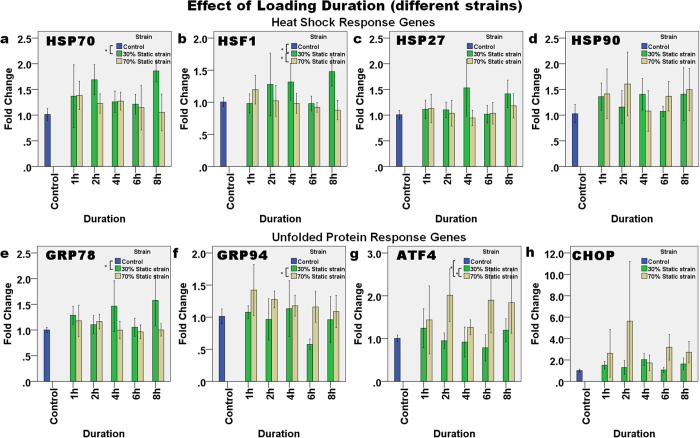
The effect of loading duration and different compressive loading strains (30% and 70% static strain) on the relative level of gene expression. The HSR genes: (**a**) HSP70, (**b**) HSF1, (**c**) HSP27, and (**d**) HSP90, and UPR genes: (**e**) GRP78, (**f**) GRP94, (**g**) ATF4, and (**h**) CHOP demonstrated different expression patterns for the different loading strains. Asterisks indicate statistically significant differences (p < 0.05). In each case, the mean ± 2SE fold change of gene expression was normalized to the control without loading. n = 3 (30%) and n = 6 (70%).

Figure 5 shows the effect of changing the loading duration at static strains of 30% and 70% on the expression levels of the HSR and UPR genes. HSP70 (Fig. 5a) was upregulated slightly to ~1.5-fold during static loading. Two-way ANOVA showed that the loading strain significantly affected the upregulation of HSP70 (p = 0.015), where the difference was between the 30% strain group and the control group (p = 0.006) but not other pairs. A similar trend was also found with the expression of HSF1 (Fig. 5b), which was also upregulated and downregulated at static strains of 30% and 70%, respectively, and both in a linear manner with the change in loading duration (p = 0.015, R^2^ = 0.193). Two-way ANOVA showed that the loading strain affected the expression of HSF1 (p = 0.003), and that there was an interaction between loading duration and strain strength in affecting HSF1 expression (p = 0.006). The *post hoc* tests revealed that with a static strain of 30%, the expression of HSF1 was significantly different from that in the control (Dunnett’s *t*-test, p = 0.047) and in the 70% static strain group (Bonferroni’s test, p = 0.010). Regarding the UPR genes, two-way ANOVA revealed that a combination of duration and loading strain affected the expression of GRP78 (Fig. 5e, p = 0.035), and loading strain alone affected the expression of GRP78 (p = 0.002). Furthermore, Dunnett’s T3 *post hoc* test conducted after one-way ANOVA showed that at 30% strain, GRP78 expression was significantly different from the control (p = 0.011) but not between other pairs. In contrast, the observed upregulation in GRP94, ATF4 and CHOP appeared to be higher during 70% static strain than other groups and this effect of strain was significant in expression of both GRP94 (two-way ANOVA, p = 0.002), and ATF4 (p = 0.002). Bonferroni’s *post hoc* tests showed that at 70% strain the level of expression was significantly higher than at 30% strain for GRP94 (p = 0.010) and ATF4 (p = 0.001), as well as higher than the control (p < 0.001) for ATF4.

#### Effect of incubation duration (with different types of loading)

**Figure 6:**
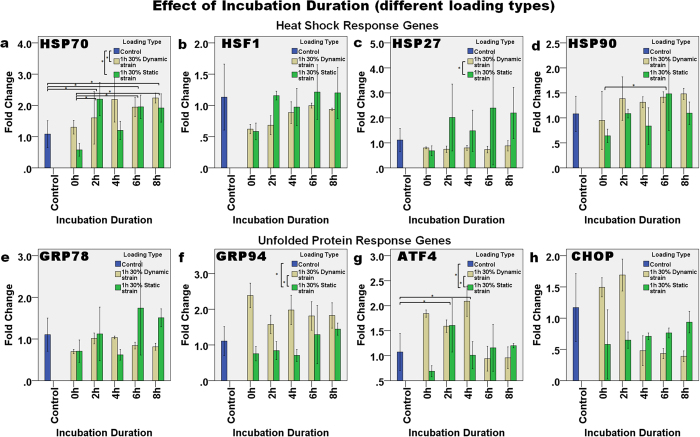
The effect of incubation duration after one hour of either dynamic or static loading on the relative level of gene expression. The expression of the HSR genes: (**a**) HSP70, (**b**) HSF1, (**c**) HSP27, and (**d**) HSP90, and UPR genes: (**e**) GRP78, (**f**) GRP94, (**g**) ATF4, and (**h**) CHOP continued to change with the incubation duration after load removal. Asterisks indicate statistically significant differences (p < 0.05). In each case, the mean ± 2SE fold change of gene expression was normalized to the control without loading. n = 3 for each sample.

The effect of incubation duration after loading was analyzed to investigate whether there is a transient or delayed expression of the genes after the load is removed. Figure 6 shows gene expression immediately (i.e., at 0 h), and then 2 h, 4 h, 6 h and 8 h after 30% dynamic or static strain, relative to the control. The expression of HSP70 (Fig. 6a) and HSF1 (Fig. 6b) increased in a linear manner with incubation duration after 30% dynamic strain (p = 0.022, R^2^ = 0.343 and p < 0.001, R^2^ = 0.630, respectively). The expression of HSF1 also increased linearly with incubation duration after 30% static strain (p = 0.047, R^2^ = 0.270). The expression of HSP70 was continually upregulated up to ~2-fold, from 2 h to 8 h after loading. Two-way ANOVA showed that there was significant interaction between incubation duration and loading type in affecting HSP70 expression (p = 0.031), and Bonferroni’s *post hoc* test demonstrated that the control was significantly different from incubation durations of 2 h (p = 0.043), 6 h (p = 0.026) and 8 h (p = 0.003). An incubation duration of 0 h was also significantly different from those of 2 h (p = 0.010), 6 h (p = 0.006) and 8 h (p = 0.002). Similarly, the expression of HSP90 (Fig. 6c) was significantly affected by the incubation duration (p = 0.031) and loading type (p = 0.033), but the combined effect was not significant (p = 0.698). Bonferroni’s *post hoc* test showed that the expression of HSP90 increased significantly 6 h after loading, when compared with immediately (0 h) after loading (p = 0.041). The expression levels of GRP94 (Fig. 6f), ATF4 (Fig. 6g) and CHOP (Fig. 6h) were all upregulated immediately after dynamic loading and then they slowly decreased over time, whereas For ATF4, the effects of incubation duration alone were also shown to be significant (p = 0.008), and the *post hoc* test showed that the expression level in the control was significantly different from that at 2 h (p = 0.024) and 4 h (p = 0.045). After static strain, the expression levels of GRP94 (p = 0.010, R^2^ = 0.407) and CHOP (p = 0.046, R^2^ = 0.272) increased linearly with incubation time. In contrast, after dynamic strain, the expression of ATF4 (p = 0.003, R^2^ = 0.514) and CHOP (p < 0.001, R^2^ = 0.715) decreased linearly with incubation duration. These results therefore indicate that the loading type was a significant factor in regulating the expression of HSP70 (two-way ANOVA, p = 0.001), HSP72 (p = 0.002), GRP94 (p < 0.001) and ATF4 (p = 0.004). Bonferroni’s *post hoc* test showed that the upregulation of HSP70 in the dynamic strain group was significantly greater than in the control group (p = 0.003), but the difference between the control and static strain was not significant (p = 0.080), although the Dunnett’s T3 *post-hoc* test indicated that these data were significantly different (p = 0.045). The upregulation of ATF4 in the dynamic strain group was also significantly different from that in the control (p = 0.027) and static strain group (p = 0.011). Dunnett’s T3 *post hoc* test after one-way ANOVA was performed for the HSP27 and GRP94 groups due to them having unequal variances, and the results showed that the expression of HSP27 after dynamic strain was significantly different from that after static strain (p = 0.018). In addition, the expression of GRP94 after dynamic strain was different from both the control (p = 0.022) and static loading (p < 0.001) groups.

#### Effect of incubation duration (with different magnitude of strain)

**Figure 7:**
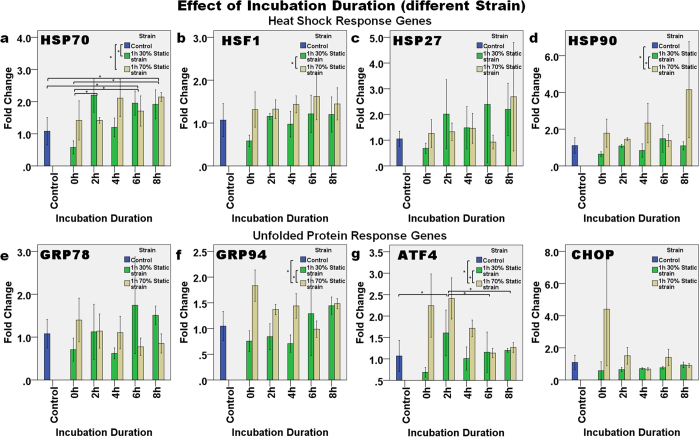
The effect of incubation duration after one hour of either 30% or 70% static loading on the relative level of gene expression. The expression of the HSR genes: (**a**) HSP70, (**b**) HSF1, (**c**) HSP27, and (**d**) HSP90, and UPR genes: (**e**) GRP78, (**f**) GRP94, (**g**) ATF4, and (**h**) CHOP continued to change with incubation duration after load removal. Asterisks indicate statistically significant differences (p < 0.05). In each case, the mean ± 2SE fold change of gene expression was normalized to the control without loading. n = 3 for each sample.

Figure 7 demonstrates the expression of genes at different incubation durations after static strain of either 30% or 70%. The level of expression of HSP70 (Fig. 7a) in general increased as the incubation duration increased. Two-way ANOVA showed that the level of expression of HSP70 was significantly affected by the incubation duration (p = 0.002), and it was also affected by the combined effect of incubation duration and loading strain (p = 0.004). Bonferroni’s *post hoc* test showed that HSP70 expression was significantly different between incubation durations of 2 h and 0 h (p = 0.023), between 6 h and the control (p = 0.045), between 6 h and 0 h (p = 0.018), between 8 h and the control (p = 0.005), and between 8 h and 0 h (p = 0.002). For the difference in loading strain, the expression of HSP70 in the control group was significantly different from that with 30% static strain (p = 0.050) and with 70% strain (p = 0.005). After 70% static strain, the expression of HSP70 increased linearly as the incubation duration increased (p = 0.032, R^2^ = 0.308).

Two-way ANOVA showed that the loading strain affected the expression of HSF1 (Fig. 7b) significantly (p = 0.003) and Bonferroni’s *post hoc* test demonstrated significant differences in the level of HSF1 expression in both the 30% and 70% strain groups (p = 0.009). Incubation duration and loading strain were also shown to significantly affect the expression of HSP90 (Fig. 7d) (p = 0.029 and p < 0.001, respectively). Dunnett’s T3 *post hoc* test showed that the expression of HSP90 in the 70% static strain group was significantly greater than in the controls (p = 0.050) and in the 30% static strain group (p = 0.020).

On the other hand, the expression of the UPR genes (Fig. 7e–h) in general decreased over time after 1 h of 70% static strain. For example, a linear decrease in the expression level of GRP78 (p = 0.020, R^2^ = 0.351) and ATF4 (p < 0.001, R^2^ = 0.635) was found with an increase in the incubation duration following 70% static strain. Two-way ANOVA showed that the combined effect of incubation duration and loading strain had a significant effect on the expression of GRP78 (p = 0.010), GRP94 (p = 0.003), ATF4 (p = 0.005) and CHOP (p = 0.007). The expression of GRP94 was also significantly affected by the loading strain (p = 0.001), and Bonferroni’s *post hoc* test showed that the expression of GRP94 after 70% static strain was significantly different from the controls (p = 0.041) and from the 30% static strain group (p = 0.002). In addition, the expression of ATF4 was also significantly affected by both the loading strain (p < 0.001) and duration (p = 0.003). Bonferroni’s *post hoc* test found that the expression of ATF4 in the 70% static strain group was different from the controls (p = 0.001) and from the 30% strain group (p < 0.001). The expression of ATF4 also increased significantly at 2 h after loading, when compared with the control (p = 0.002), 6 h (p = 0.004) and 8 h (p = 0.012) groups.

#### Effect of incubation duration (at different loading durations under high strain)

**Figure 8:**
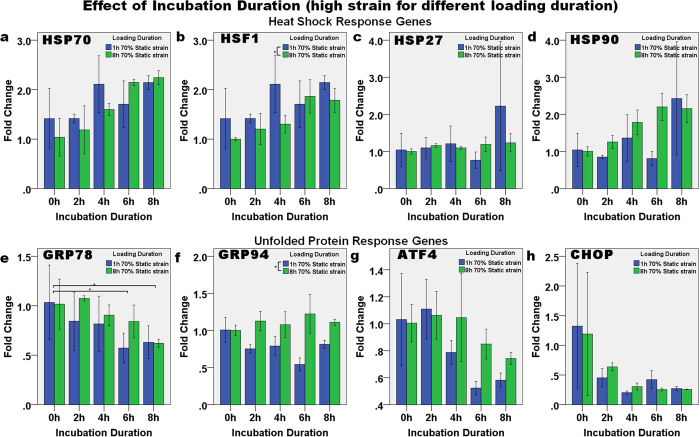
The effect of incubation duration after one or eight hours of loading at 70% static strain on the relative level of heat shock response genes. (**a**) HSP70, (**b**) HSF1, (**c**) HSP27, and (**d**) HSP90, and UPR genes: (**e**) GRP78, (**f**) GRP94, (**g**) ATF4, and (**h**) CHOP. The expression level of all the HSR genes except HSP27 increased with incubation duration after load removal. In contrast, expression level of all the UPR genes except GRP94 decreased with incubation duration after load removal. Asterisks indicate statistical difference (p < 0.05). In each case, the mean ± 2SE fold change of gene expression was normalized to the control without loading. n = 3 for each sample.

In order to determine if NPCs are under stress (i.e., they have a high degree of matrix deformation) when they are under high strain, 70% static strain was applied for either 1 h or 8 h. The expression of the HSR and UPR genes was then normalized to the 0 h samples in order to analyze the direct effect of incubation duration after strain (Fig. 8). The expression levels after high static strain (70%) were tightly associated with the post-loading incubation duration. A similar trend was observed at both loading durations used. The HSR (Fig. 8a–d) and UPR (Fig. 8e–h) genes showed a contrasting trend of expression. After 1 h strain, increasing the duration of incubation upregulated the expression of HSP70 (p = 0.031, R^2^ = 0.311) and HSF1 (p = 0.031, R^2^ = 0.311) in a linear manner. However, after 8 h static strain the expression of HSR genes fitted the linear relationship with incubation duration better: HSP70 (p < 0.001, R^2^ = 0.811), HSF1 (p < 0.001, R^2^ = 0.693), HSP27 (p = 0.041, R^2^ = 0.284) and HSP90 (p < 0.001, R^2^ = 0.776). In contrast, the expression of GRP78 (p = 0.016, R^2^ = 0.370), ATF4 (p = 0.001, R^2^ = 0.608) and CHOP (p = 0.027, R^2^ = 0.324) decreased in a linear manner with incubation duration after both 1 h and after 8 h static strain: GRP78 (p = 0.001, R^2^ = 0.592), ATF4 (p = 0.024, R^2^ = 0.334) and CHOP (p = 0.009, R^2^ = 0.423).

### Immunofluorescence staining of HSP70

**Figure 9:**
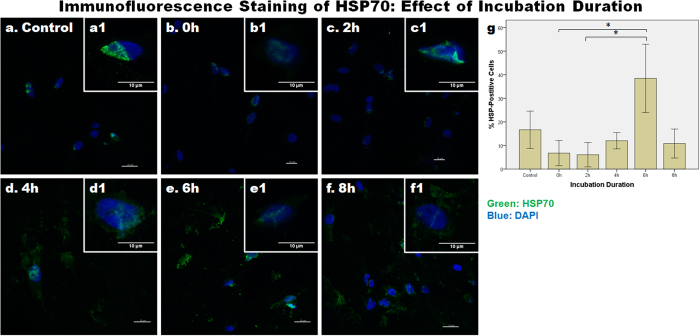
The effect of incubation duration after loading on the expression of HSP70 protein. Representative images from (**a**) control and (**b–f**) various incubation durations following one hour of 70% static strain are shown. (a1–f1) The inserts show magnified views of the nuclei. Sections were immuno-labeled with an anti-HSP70 antibody and the nuclei were labeled with DAPI. Scale bars: 10 μm. (**g**) Bar chart to show the mean ± SEM percentage of HSP70-positive cells (i.e., calculated from the number of HSP70-positive cells and the total number of cells) at different incubation durations. n = 10 images in each experimental group. The asterisks indicate statistically significant differences (p < 0.05).

Figure 9 shows images of cells that were fixed and immunolabeled with an anti-HSP72 (inducible form of HSP70) antibody following various different incubation durations after loading. When compared with the control, the level of HSP70 decreased within 2 h after loading (Fig. 9b,c); then between 2 h and 4 h after loading, the level of HSP70 increased (Fig. 9d); the level peaked at 6 h after loading (Fig. 9e), and then decreased by 8 h after loading (Fig. 9f). At 2 h to 8 h after loading, HSP70 was found to be localized outside the nucleus, possibly in the cytoplasm. Quantification of the percentage of HSP70-positive cells, also demonstrated a similar pattern, such that the percentage of positively-labeled cells decreased within 2 h after loading, and then increased to about 40% at 6 h after loading (Fig. 9g). One-way ANOVA showed that the difference in HSP70 expression due to incubation duration was significant (p < 0.001), whereas Dunnett’s T3 test showed that the 6 h post-loading group was significantly different from the 2 h (p = 0.012) and 4 h (p = 0.018) groups.

## Discussion

The intervertebral disc cells appeared to adapt to their change in environment (i.e., from being in a monolayer to being encapsulated in a 3D collagen matrix). One day after encapsulation, an increase in the expression of HSP70 indicated that cells were exhibiting a stress response, however thereafter, the expression of HSP70 began to gradually decrease, suggesting that the cells might have started to adapt to the environment. The reason why HSP70 is upregulated in 3D culture is not well understood, although, as previous studies indicate, the upregulation of stress response genes might be some form of cell survival mechanism^19^,^20^. Low levels of apoptosis in the initial phase after collagen encapsulation have also been reported previously^18^,^21^, which again indicates that the surviving cells might activate the stress response as a protection mechanism. Alternatively, the cellular and biochemical changes that take place when cells are detached from a monolayer and then encapsulated in extracellular matrix might also induce cell death. HSP70 is known to be anti-apoptotic by interfering or inhibiting the activation of pro-apoptotic factors^1^,^22^,^23^. Hence, the remaining cells might survive by upregulating HSP70. Another possible reason for the upregulation of apoptosis might be that the contraction of the collagen construct imposes a certain amount of mechanical stress on the cells. Contraction is commonly observed in cells encapsulated in collagen, and it is thought to be mediated via integrins^24^. Such a stress response was reported in human mesenchymal stem cells (MSCs) seeded on collagen films and it was suggested to be induced by the contraction of the collagen I matrix via integrins^25^. The reason why a stress response occurs on cell encapsulation in collagen needs further investigation.

Our results indicated that mechanical loading leads to a mild stress response genes upregulation in NPCs. The expression of HSR and UPR genes changes with the different conditions of loading. The effect of compression on the stress response genes is not strong immediately after a short duration of loading although there is difference due to the loading frequency in HSP70. However, the expression of stress response genes was on the whole higher in the 70% static strain group than in the 30% static strain group when the cells were compressed for a longer duration and after the removal of the compression loading; this is especially so for HSP70, HSF1 and ATF4. In addition, the expression of genes in the static strain group were also higher than in the dynamic strain group. Differences in loading type have been reported previously, where the expression of HSP70 in a chondrocyte cell line was upregulated with an increased duration in static pressure but not with cyclic pressure^2^. The lower stress response observed in the dynamic loading group might be because this type of loading allows the cells to recover in between each loading.

Notably, the upregulations were not in strong association with loading duration but incubation duration in the current study. The results suggest that at high level of static strain, cellular response would continue even after the load is removed. The effect of incubation time after loading on the expression of stress response genes has also been observed in tendon fibroblasts, such that there was higher HSP70 protein expression at 2 hours and 8 hours after 1-h loading^22^. It was suggested that the increase might be due to an acute response to stress^7^, and a global increase in protein synthesis in order to replace damaged proteins followed^26^. In addition to cell survival and protein synthesis, loading induced stress response was suggested to be a downstream event of the mechanotransduction pathway, where stretch-activated ion channels were demonstrated to upregulate HSP70^27^. Integrins, another group of important mechanosensors were also suggested to be coordinated with cellular stress response^28^. The mechanism and pathways that lead to cellular stress response needs to be elucidated in future experiments.

Interestingly, over time the expression of HSR genes increased whereas that of UPR genes decreased. A high static strain applied for a longer duration, results in the elevated expression of UPR genes, which suggests that under extreme stress the cells might activate the UPR. On load removal, the HSR genes continued to be upregulated to protect the cells, whereas the UPR genes were downregulated, indicating that cells were inhibiting the endoplasmic reticulum (ER) stress pathway, which usually leads to apoptosis. However, the ER stress experienced by cells may be minimal as the upregulation in UPR genes especially GRP78 are mild. Furthermore, XBP1 splicing was not observed (Supplementary Fig. S3), which demonstrates that IRE1α\XBP1pathway was not involved. Proteins expression for the HSR and UPR pathways would need to be examined to further elucidate the process.

The heat shock response is generally considered to be involved in repair and survival mechanism of cells^1^,^29^. HSP70 is known to have anti-apoptotic properties by interfering or inhibiting the activation of caspase^1^,^22^,^23^. In addition, mechanical loading-induced apoptosis is suggested to be mediated through nitric oxide signaling^30^,^31^ and is associated with ER stress^11^. This confirms another study, which demonstrated that in anulus fibrosus cells, the ER pathway was activated in loading-induced apoptosis, but when CHOP expression was silenced then the cells were rescued from apoptosis^17^. In the current study, cell death did not increase after loading for a short duration Expression of HSR genes, especially HSP70, suggests that cells express these genes as a form of cell survival mechanism to prevent apoptosis. Our results are supported by a previous study, where it was shown that in human tendon fibroblasts, repetitive loading for 2 days resulted in an increased rate of apoptosis and an upregulation of HSP70^8^. In addition, HSP70 was not expressed in apoptotic cells after loading^5^. These two studies confirm that cells expressing HSP70 do not undergo apoptosis and that apoptotic cells do not express HSP70 after loading.

Heat shock proteins expression had been associated with disc degeneration, pathology and aging^12^,^13^. The association was suggested to be related to mechanical and environment stress experienced by the cells, where mechanical stimulation is also one of the risk factors for disc degeneration^32^,^33^. In addition to that, mild disc degeneration is also associated with cell apoptosis through ER pathway^16^. Our results provide support for this suggestion by demonstrating expression of HSR genes and UPR genes in response to compressive loading. Understanding loading induced stress and subsequent changes might enable us to better understand pathoetiology of disc degeneration.

Our results indicate that healthy NPCs might activate the relevant stress response to cope with mechanical stress. When investigating the effect of mechanical loading on the stress response of NPCs, a comparison between cells in healthy and degenerative discs will provide us with further insights regarding the mechanisms involved in the development of disc degeneration. Indeed, a similar study that was conducted to investigate the role of integrin signaling in IVDs during mechanical stimulation, found that the mechanosensing signaling pathway is altered in NPCs from degenerated discs^34^. Similar findings were found during matrix remodeling in both NPCs and AFCs^35^,^36^,^37^. In the current study, we demonstrated that HSP70 and HSP90 are upregulated more in AFCs than in NPCs (Supplementary Fig. S4). Furthermore, the similar changes in expression of both the HSR and UPR genes due to loading were found to be related to age (Supplementary Fig. S5). This piece of information can be used to further investigate the responses of different cell types, especially when comparing the effect of mechanical loading on healthy and diseased NPCs. The difference between NPCs from healthy and degenerative discs in terms of their stress response may be used to define how a healthy disc should function under loading.

In this study, we investigated the effect of mechanical stress on the stress response of NPCs. In our *in vitro* culture model, the encapsulation of NPCs in collagen was itself found to induce a transient stress response, as shown by the transitory upregulation of HSP70. However, different types of mechanical loading further induced the stress response in NPCs in varying ways. In addition, the upregulation of the HSR genes and downregulation of UPR genes during post-loading (high strain) incubation suggests that the stress response genes play a role in cell survival and protein synthesis, as cell death under high strain was not observed. Our results provide an explanation about how mechanical stress might induce and control the expression of HSR and UPR genes in NPCs.

## Methods

### Cell isolation and culture

Bovine tails of one- to two-year-old cattles were purchased from a local market within 12 hours after slaughtering and hence no ethical approval was needed. The caudal disc tissues were dissected and digested in 0.25% pronase (Sigma, St. Louis, MO, USA) for one hour and in then 600 U/ml collagenase (Sigma) for 16 hours at 37 °C. The enzymes were prepared in Dulbecco’s Modified Eagle’s Medium (DMEM, Gibco, Grand Island, NY, USA). The cells were then collected using a 40 μM cell strainer and centrifuged at 800 g for 10 min. Cells were further washed with DMEM twice before counting. Cells were seeded in a 100 mm culture dish at 5 × 10^5^ cell density and expanded until they were at 80% to 90% confluence. DMEM-LG supplemented with 10% fetal bovine serum (Gibco) and 1% penicillin/streptomycin (Gibco) was used as culture media. Cells were then either subcultured, or else frozen down in media comprised of 10% DMSO and 90% culture media prior to further use.

### Cell encapsulation in collagen construct

Collagen microencapsulation was used in this study as the 3D *in vitro* culture model. This system has previously been shown to be able to preserve the morphology and phenotype of rabbit NPCs^18^. Cells at passage 2 were thawed and expanded in a monolayer using a 100 mm culture dish for three days until at 70–80% confluence. Cells were then detached using 0.25% trysin/EDTA (Gibco) and encapsulated in a collagen construct, as described previously^21^,^38^. In brief, rat tail collagen type I (BD Biosciences, San Jose, CA, USA) was neutralized with 1N NaOH and diluted with culture medium before being mixed with the cells, after which 50 μL aliquots of the mixture were transferred into cylindrical molds and incubated in a humidified incubator at 37 °C and 5% CO_2_ for 1 h to form disc-shaped gels. The molds were fabricated by cutting 1 ml syringes into short hollow cylindrical tubes. Each construct consisted of 2 mg/ml collagen and 25,000 cells. Following gel formation, the constructs were covered with culture medium and allowed for contract for 4 days.

### Compressive loading

To study the effect of loading, a micromanipulator-based loading device was used, as described previously^38^,^39^,^40^. In brief, the disc-shaped constructs were transferred into a two-compartment Petri dish and covered with culture media. The Petri dish was placed onto an inverted microscope (DMIRB, Leica Microsystems, Wetzlar, Germany) with a heated plate set to 37 °C. The constructs were placed vertically in between a custom-made spatula and the partition wall of the Petri dish such that the thickness of the constructs could be readily observed under the microscope. The spatula was connected to a micromanipulator (MP285, Sutter Instruments, Novato, CA, USA), which could be programmed to compress the constructs. A preloading force was initially applied to reduce the construct thickness by 10%, after which specific loading strains were applied to compress the constructs during each experiment. Constructs without loading were placed in the same culture medium and used as controls.

In order to test the effect of loading duration, constructs were compressed for 1 h, 2 h, 4 h, 6 h or 8 h under various different loading types and strains. To test the effect of incubation duration after loading, constructs were incubated in culture media for 0 h, 2 h, 4 h, 6 h or 8 h after loading was applied.

### Cell viability

The viability of cells after loading was assessed using a Live/Dead Viability Kit (Molecular Probes, Eugene, OR, USA) according to the manufacturer’s instructions. In brief, constructs were incubated in 2 μM calcein AM and 4 μM ethidium homodimer-1, diluted with serum-free culture medium for 45 min at 37 °C in the dark. Constructs were then washed with phosphate buffered saline (PBS) and visualized under a Nikon TE2000-U florescence microscope (Tokyo, Japan).

### Terminal deoxynucleotidyl transferase (TdT) dUTP nick-end labeling (TUNEL) assay

The terminal deoxynucleotidyl transferase (TdT) dUTP nick-end labeling (TUNEL) assay was conducted on the samples that had been subjected to 70% static strain by using an *in situ* cell death detection kit with fluorescein (Roche, Basel, Switzerland), according to the manufacturer’s instructions. In brief, tissues were fixed using buffered 4% paraformaldehyde and then cryo-sectioned. The sections were then hydrated with PBS and incubated with permeabilisation solution (0.1% Triton X-100 in 0.1% sodium citrate, both Sigma) for 2 min on ice. Sections were then washed twice with PBS before they were incubated with the TUNEL reaction mixture. The TUNEL reaction mixture was freshly prepared by mixing Label solution and Enzyme solution in a 1:10 ratio. Positive controls were prepared by incubating sections with DNase I (Sigma) for 10 min at room temperature to induce DNA strand breaks. DNAase I was prepared at a concentration of 3 U/ml in 50 mM Tris-HCL and 1 mg/ml bovine serum albumin (BSA, Jackson Immuno Research, West Grove, PA) at pH 7.5. Sections incubated in Label solution without Enzyme solution were used as negative control. Slides were then rinsed with PBS three times before being mounted with Fluoro-Gel II containing DAPI (EMS, Hatfield, PA, USA). Sections were visualized using the Nikon TE2000-U fluorescence microscope at 20X magnification. Images of three field of view were acquired per section and two sections were used per sample for quantification. In each field of view, the cells generating green fluorescence were quantified. The total number of cells was then determined by counting the DAPI-labeled nuclei. The number of green fluorescence cells was then normalized to the total number of calls in order to calculate percentage of TUNEL-positive cells.

### Gene expression

Total RNA was extracted by using TRI reagent. In brief, cell-encapsulated collagen constructs were dissolved in TRI reagent (Molecular Research Center Inc., Cincinnati, OH, USA). The RNA was extracted using 1-bromo-3-chloropropane (Sigma), after which it was precipitated using isopropanol (Sigma) and washed with 75% ethanol. The quality and quantity of RNA were checked using a NanoDrop2000 spectrophotometer (Thermo Fisher Scientific Inc., Waltham, MA, USA). Reverse transcription was carried out with a High-capacity Reverse Transcription Kit (Applied Biosystems Inc. (ABI), Foster City, CA, USA). Oligonucleotide primers for quantitative PCR (qPCR) were either designed by Beacon Designer™ (Premier Biosoft, Palo Alto, CA, USA) based on sequences from the NCBI GenBank database, or else they had been designed and published by others^41^,^42^. Information about the primers used, is presented in Table 1. qPCR was performed with a STEPONE Plus PCR system (ABI). Every reaction consisted of 3 μL water, 5 μL Power SYBR Green Super mix (ABI), 1 μL primers (0.25 μM) and 1 μL cDNA. All C_t_ values of the genes were normalized to the C_t_ value of ribosomal 18S RNA. In addition, the fold change in gene expression was calculated relative to the control group (without loading) by the 2^−ΔΔCt^ method.

### Immunofluorescence Staining

Tissues were fixed in 4% buffered paraformaldehyde, after which they were frozen and then cryosectioned for immunofluorescence staining. Sections of 40 μm depth were stained with a mouse monoclonal anti-HSP72 (i.e., the inducible form of HSP70, at 1:100 dilution, ADI-SPA-810, Stressgen, Enzo, NY, USA). Briefly, sections were hydrated in PBS and cells were permeabilized with 0.5% Tween-20. Blocking was done using 3% BSA for 30 min. Sections were then incubated in the anti-HSP72 antibody at 4 °C overnight. After washing with PBS, sections were then incubated in the appropriate fluorochrome-conjugated secondary antibody (at a 1:400 dilution) for one hour. They were then mounted with Fluoro-Gel II containing DAPI. Images were acquired using a Carl Zeiss LSM700 confocal scanning microscope (Jena, Germany). To quantify the percent of HSP70-positive cells, images were taken using the Nikon TE2000-U fluorescence microscope at 20X magnification. Images were acquired at 10 different locations for each sample, after which the number of HSP70-positive cells and the total number of cells (identified via the DAPI staining) were then counted. Data was presented as % HSP70-positive cells.

### Statistical analysis

All statistical analyses were carried out using SPSS 19.0 (IBM, Armonk, NY, USA). The significance level was set to 0.05. Results were tested for normality assumption by a one-sample Kolmogorov–Smirnov test. One-way or two-way ANOVA with Bonferroni or Dunnett’s T3 *post hoc* tests were used to determine significant differences among the multiple groups.

## Additional Information

**How to cite this article**: Chooi, W. H. and Chan, B. P. Compression loading-induced stress responses in intervertebral disc cells encapsulated in 3D collagen constructs. *Sci. Rep.* **6**, 26449; doi: 10.1038/srep26449 (2016).

## Supplementary Material

Supplementary Information

## Figures and Tables

**Table 1:** Primers used for the quantitative real time PCR *in vitro* study.

Gene	Forward Primer (5′-3′)	Reverse Primer (5′-3′)
18S	ACG GAC AGG ATT GAC AGA TTG	CCA GAG TCT CGT TCG TTA TCG
HSP70	AGG AGG TGG ATT AGG AAT	GGA CAG TTC AAC ATC TCA
HSF1	GCA GGT GTT CAT AGA ATT GTA TT	CTG GCT CAT CGG TCT GTT
HSP90	TTG GCT ATC CCA TCA CTC	TTC TAT CTC GGG CTT GTC
GRP78	GCA TCG ACC TGG GTA CCA CCT A	CCC TTC AGG AGT GAA AGC CAC A
GRP94	AAG AAC CTG CTG CAT GTC ACA GA	TGG CCA TCT TCT TGT GCC TCA
ATF4	CTG GAG AGA AGA TGG TAG CAG CAA	GCC CTC TTC TTC TGG CGG TA
CHOP	GAA CCT GAG GAG AAGA GTG TTC CA	AGT GAC TCA GCT GCC ATC TCT GT

The primer sequences of GRP78, GRP94, and ATF4 were obtained from^42^, whereas that of CHOP was obtained from^41^.
