# Supplementary material for: Compression loading-induced stress responses in intervertebral disc cells encapsulated in 3D collagen constructs
Source: Sci Rep. 2016 May 20;6:26449. doi: 10.1038/srep26449 (PMC4873809; doi:10.1038/srep26449)
Supplement: Supplementary Information [file srep26449-s1.pdf]

Compression loading-induced stress responses in intervertebral disc cells encapsulated in 3D collagen constructs

Wai Hon Chooi, Barbara Pui Chan*

Tissue Engineering Laboratory, Department of Mechanical Engineering, The University of Hong Kong, Pokfulam Road, Hong Kong Special Administrative Region, China.

*Corresponding author: bpchan@hku.hk

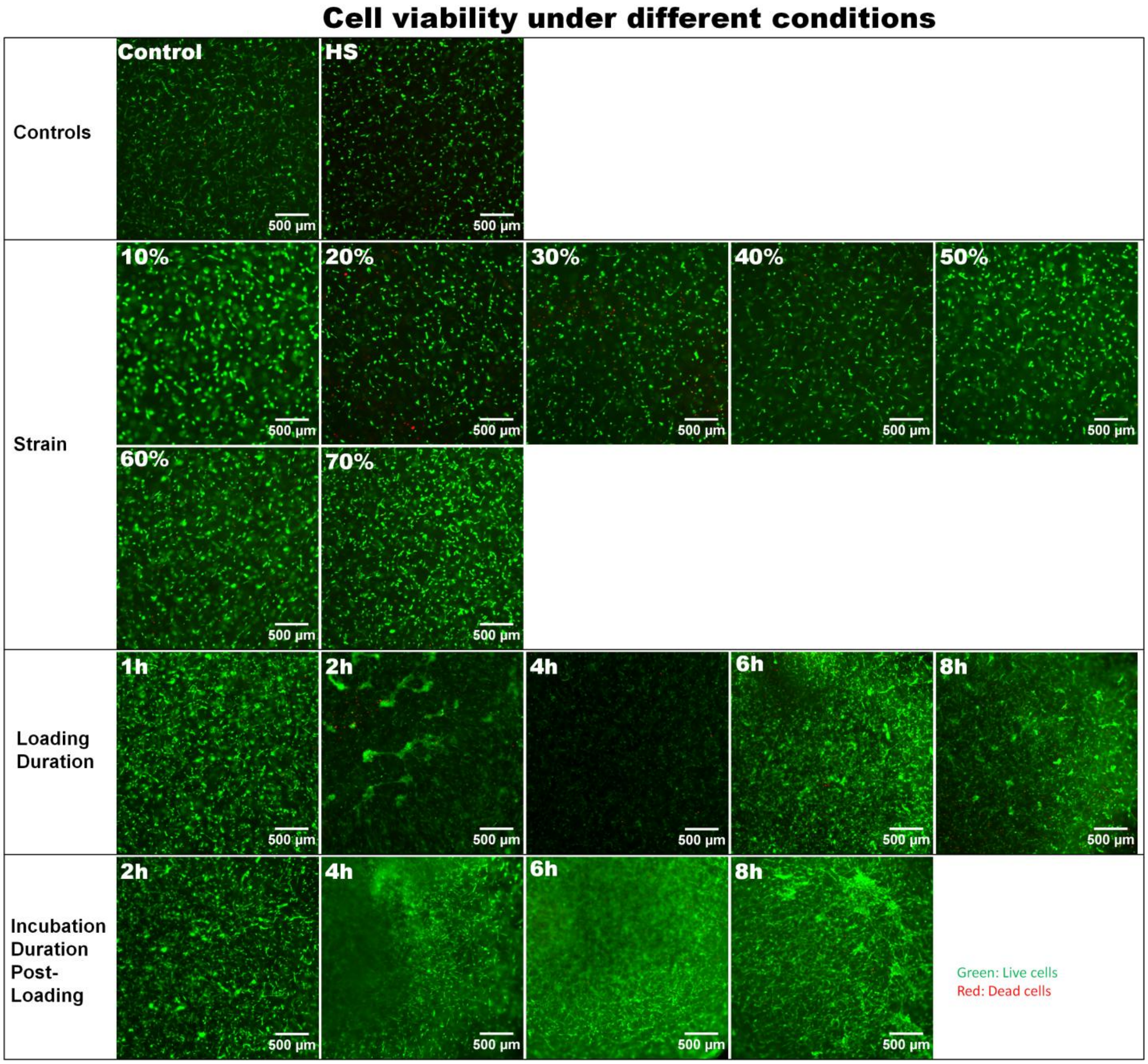

Supplementary Figure 1. Cell viability under different conditions. Viability of the NPCs after static strain in different strain, loading duration and incubation duration was assessed by Live/Dead cell viability assay. Live cells were stained with green fluorescent calcein-AM and dead cells are indicated by red fluorescent ethidium homodimer-1 staining. Scale bar: 500 µm.

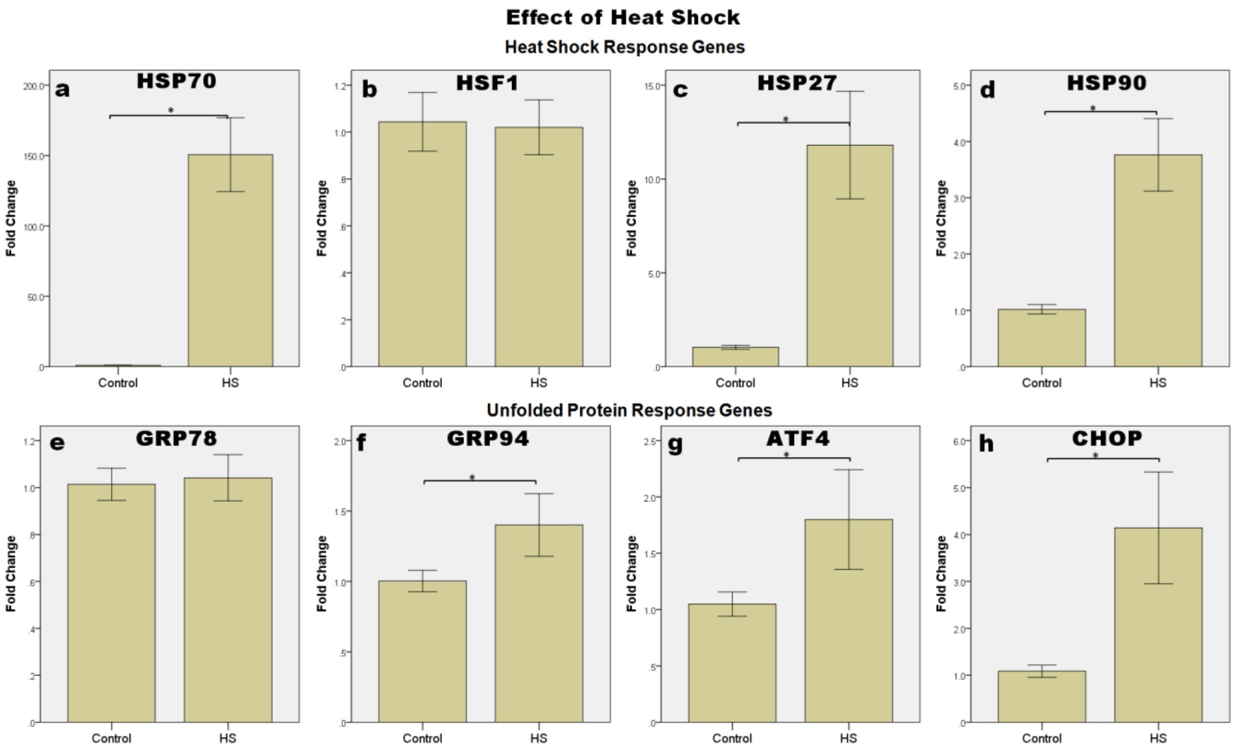

Supplementary Figure 2. Effect of heat shock on NPC expression of HSR genes (a-d) and UPR genes (e-h). Heat shock induced upregulation in HSP70 (a), HSP27 (c), HSP90 (d), GRP94 (f), ATF4 (g) and CHOP (h). Gene expressions in mean ± 2SE were normalized to 18S rRNA and relative to control. HS: Heat shock. * = statistical difference ($p < 0.05$). n = 28 for control and n = 22 for HS.

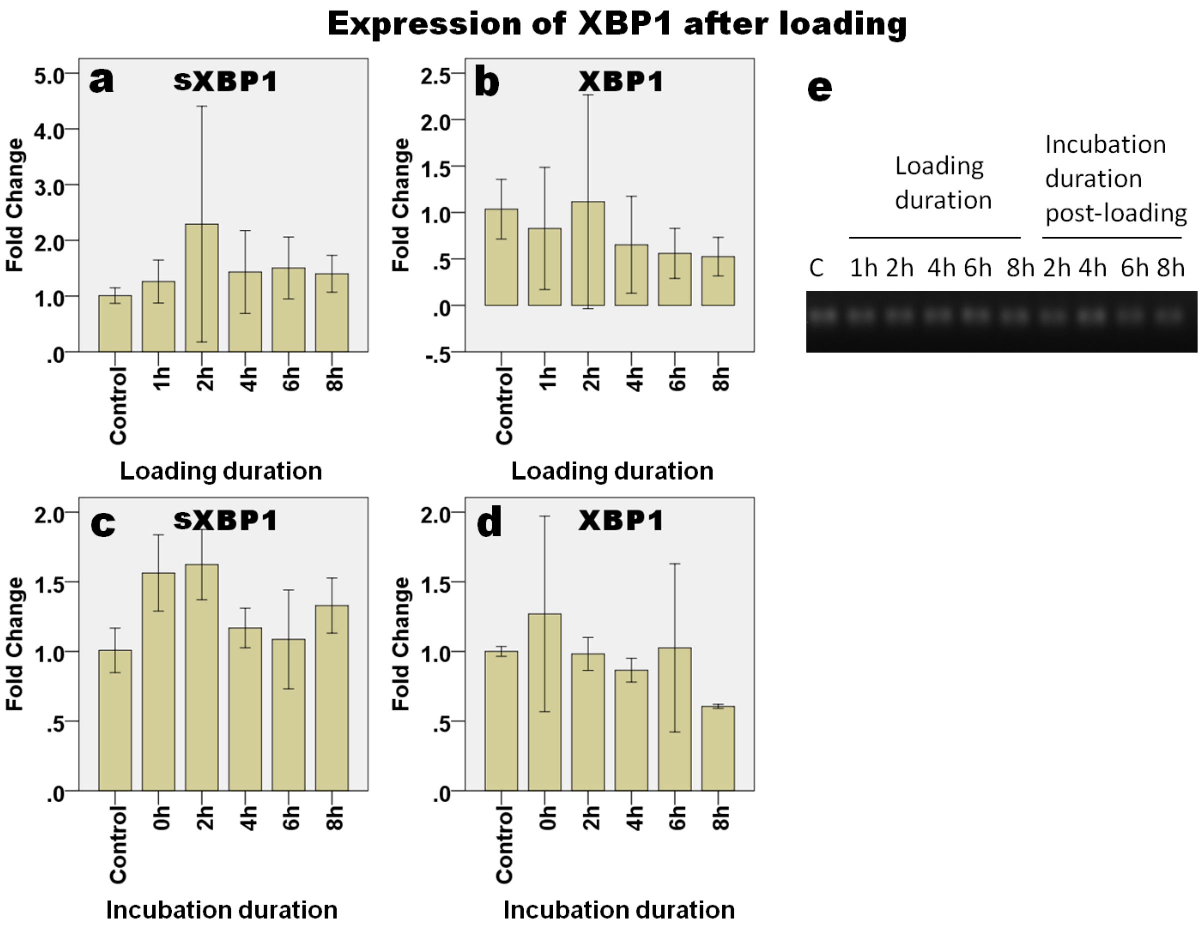

Supplementary Figure 3. Effect of loading on gene expression of XBP1 and splicing after 70% static strain for 1 h. Expression of spliced XBP1 (a,c) and total XBP1 (b,d) did not change significantly with loading duration (a,b) and incubation duration post-loading (c,d). Samples Expressions were normalized to control without loading and presented in mean ± SEM. n = 3 for each sample. Splicing was not observed in the PCR product of total XBP1 (e).

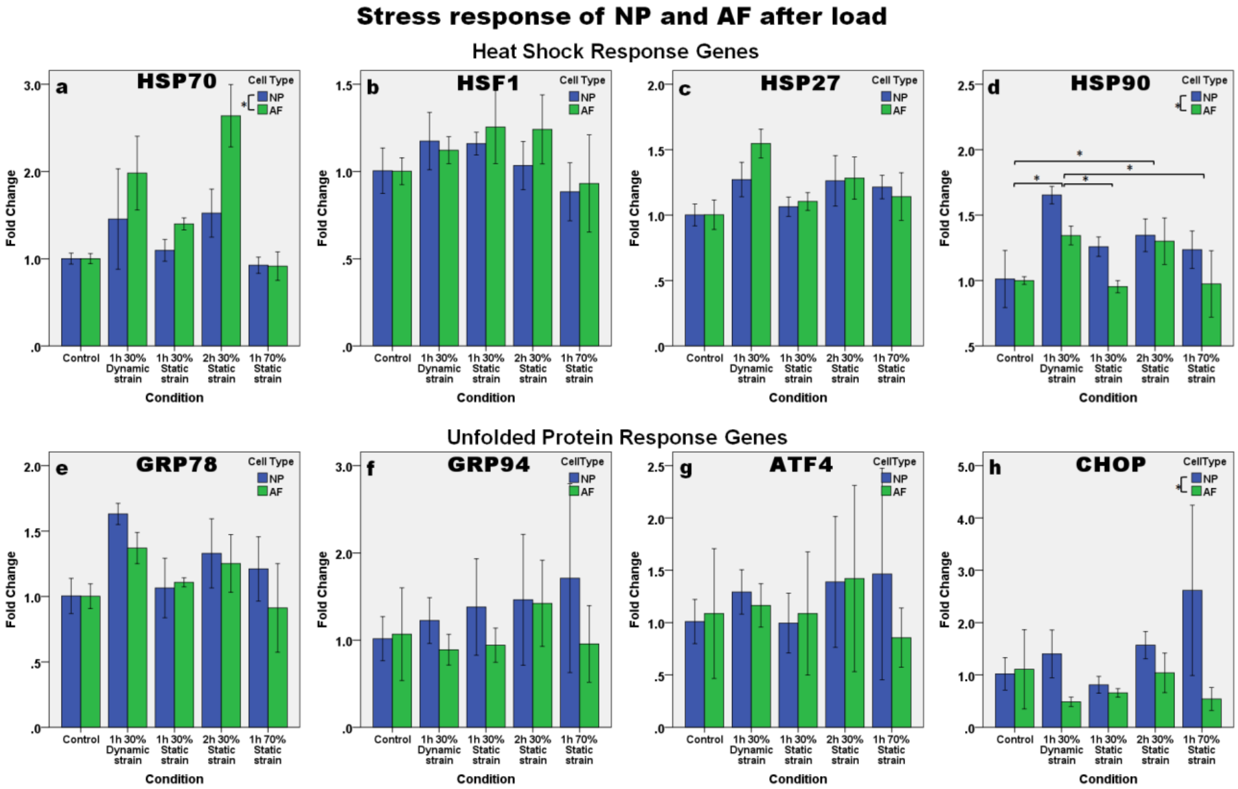

Supplementary Figure 4. Effect of loading on gene expressions of heat shock response genes (a: HSP70, b: HSF1, c: HSP27, d: HSP90) and unfolded protein response genes (e: GRP78, f: GRP94, g: ATF4, h: CHOP) was compared in NPCs and AFCs. Different loading conditions were applied. Samples were collected two hours after loading. Expressions were normalized to control without loading and presented in mean ± 2SE. * = statistically significant difference ($p < 0.05$). Significant difference due to interaction of age and condition was also found in expression of CHOP (two-way ANOVA). n = 3 for each sample.

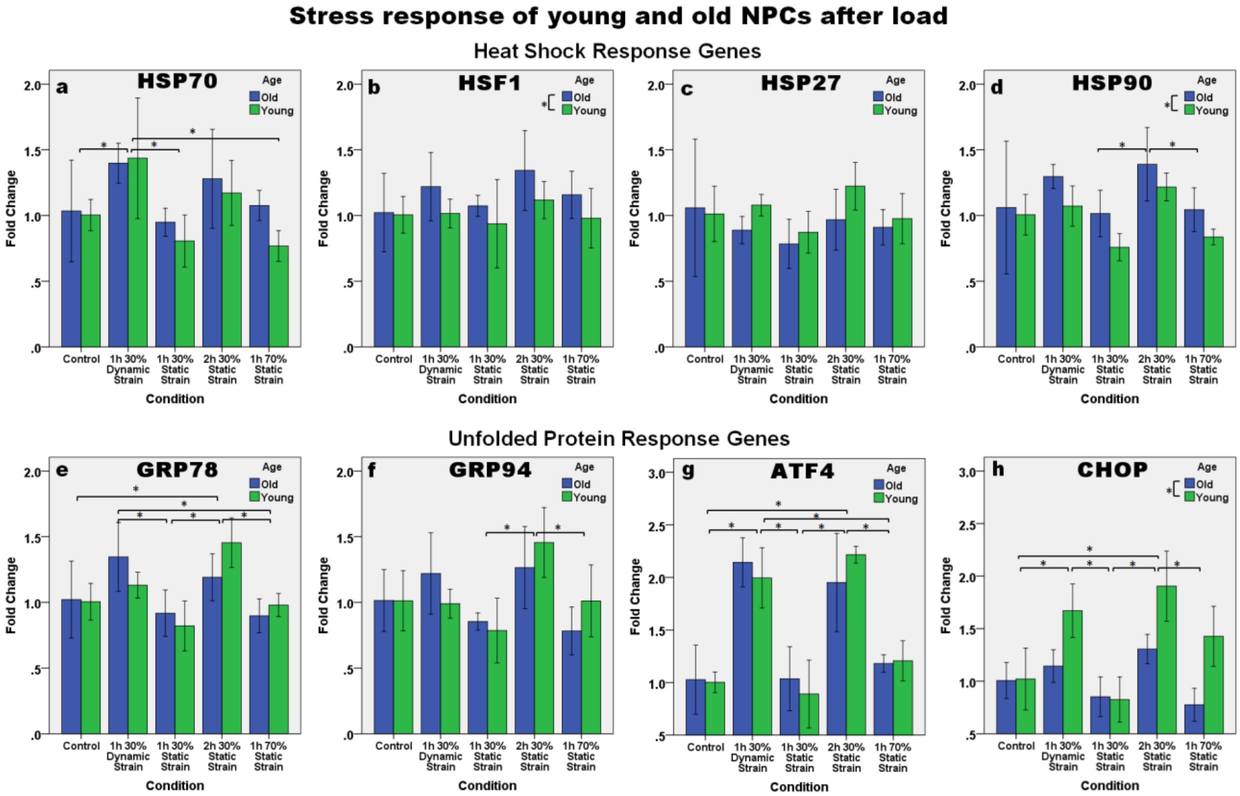

Supplementary Figure 5. Effect of loading on gene expression of heat shock response genes (a: HSP70, b: HSF1, c: HSP27, d: HSP90) and unfolded protein response genes (e: GRP78, f: GRP94, g: ATF4, h: CHOP) was compared in NPCs from old (five years old) and young (one-two years old) bovine caudal discs. Different loading conditions were applied. Samples were collected two hours after loading. Expressions were normalized to control without loading and presented in mean ± 2SE. * = statistically significant difference ($p < 0.05$). Significant difference due to interaction of age and condition was also found in expression of CHOP (two-way ANOVA). n = 3 for each sample.
